# Mitochondria and tumorigenesis: Molecular basis and therapeutic implications

**DOI:** 10.1016/j.gendis.2025.101806

**Published:** 2025-08-12

**Authors:** Chen Huang, Zichuan Xie, Jiajin Li, Chenliang Zhang

**Affiliations:** a Health Management Center, General Practice Medical Center, West China Hospital, Sichuan University, Chengdu, Sichuan 610041, China; b West China School of Medicine, Sichuan University, Chengdu, Sichuan 610041, China; c Division of Abdominal Cancer, Department of Medical Oncology, Cancer Center and Laboratory of Molecular Targeted Therapy in Oncology, West China Hospital, Sichuan University, Chengdu, Sichuan 610041, China

**Keywords:** Cell death, Drug resistance, Mitochondria, Tumor therapy, Tumorigenesis

## Abstract

Mitochondria, vital organelles within cells, govern energy metabolism. They play a pivotal role in maintaining redox homeostasis and are instrumental in the initiation and transmission of cell death signals, along with the synthesis of biological macromolecules. The role of mitochondria in tumor evolution and treatment has recently been the focus of extensive research. Studies indicate that the quality and biogenesis of mitochondria, along with their structure, functions, and macromolecule synthesis relevant to it, are intimately linked to tumorigenesis and the prognostic outcomes of clinical treatments. As such, therapies targeting mitochondria offer promising avenues to augment the efficacy of tumor treatment. We summarized the inherent links between mitochondrial structure, mitochondrial genes, metabolism of mitochondrial-related biological macromolecules, and mitochondria-regulated cell death in relation to tumorigenesis and progression. Furthermore, we reviewed the latest research progress in targeting mitochondria for tumor therapy. This study suggests that targeting mitochondria could open new avenues for developing tumor therapy.

## Introduction

Mitochondria, commonly referred to as the “powerhouses of the cell”, are essential organelles responsible for producing adenosine triphosphate (ATP),^1^^,^^2^ the chief source of energy for a variety of cellular processes. Besides their role in energy production, mitochondria are now recognized for their multifaceted involvement in cancer pathogenesis. Alterations in mitochondrial metabolism have been observed in various cancer types and are associated with tumor growth and survival.^3^ Researchers have extensively studied mitochondria-associated glucose metabolism, such as aerobic glycolysis (the Warburg effect),^4^ and have underscored the reliance of cancer cells on glycolytic pathways for energy production. In addition, aberrant mitochondrial lipid metabolism and perturbed amino acid metabolism have been implicated in tumorigenesis and tumor progression.^5^^,^^6^ Furthermore, mitochondrial DNA (mtDNA) has emerged as a key player in cancer biology. mtDNA mutations, changes in mtDNA copy number (mtDNA CN), and epigenetic modifications in mtDNA have been reported in various cancer types.^7^, ^8^, ^9^ These alterations not only affect mitochondrial function but also contribute to tumor initiation and progression.

In recent years, the interplay between mitochondria and tumor therapy has garnered significant attention. Dysregulated mitochondrial dynamics, such as increased biogenesis, impaired mitophagy, and altered fusion–fission processes, have been associated with resistance to conventional cancer therapies.^10^, ^11^, ^12^ Exploiting the unique characteristics of mitochondria, researchers have been exploring mitochondria-targeted drugs, including chemotherapeutic agents specifically targeted to mitochondria, small-molecule drugs that modulate mitochondrial function, and nanoparticles and nanomaterials for delivering mitochondria-targeted therapies, as potential therapeutics for cancer treatment.^13^

In this review, we systematically summarize the current understanding of mitochondrial structure, function, metabolism, and dynamics, and discuss the relationship between that and tumorigenesis and the development of mitochondria-targeted drugs for effective tumor therapy. By unraveling the complex interplay between mitochondria and cancer, we hope to contribute to the advancement of innovative strategies for cancer prevention and treatment.

## Structure and function of mitochondria

The “endosymbiosis theory” considers mitochondria to originate from an α-protein bacterium that is phagocytosed by eukaryotic cells.^14^ Mitochondria are maternally inherited; they vary in size and shape, ranging from 0.5 to 3 microns in size, and can be spherical, rod-shaped, or filamentous.^14^^,^^15^ Mitochondria are involved in a wide variety of biochemical processes in cells and are important sites for oxidative phosphorylation (OXPHOS), tricarboxylic acid (TCA) cycle, fatty acid β-oxidation, calcium handling, and heme biosynthesis^16^ and occupy a central position in cellular metabolism.^1^^,^^2^ Mitochondria are found in all somatic cells except mature red blood cells. The number of mitochondria in a cell varies from 1 to 10,000, generally depending on the metabolic level of the cell. The number of mitochondria in a metabolically active cell is higher.^15^

The morphology of mitochondria in the cell is not static, and they can assume different forms in different cells depending on their specific needs. This variation is achieved by mitochondrial fusion and division, which are important for cell survival and proliferation.^17^ Mitochondrial fusion helps to ensure an adequate supply of energy when cell metabolism is enhanced, while mitochondrial division plays a pivotal role in cell growth and division. These two opposing processes complement each other and work in conjunction to maintain normal cellular function. When mitochondrial division/fusion is disrupted, it can lead to a range of diseases, including cancer and neurodegenerative diseases.^3^

## Structure of mitochondria

Mitochondria can be essentially divided into four parts, from the outside to the inside: outer mitochondrial membrane, mitochondrial intermembrane space, inner mitochondrial membrane, and mitochondrial matrix.^18^ The outer mitochondrial membrane is the unit membrane located at the outermost layer of mitochondria. It not only serves as a barrier but also facilitates material exchange. It can protect cells from harmful products such as reactive oxygen species (ROS), immunogenic mtDNA, and death signals that harm mitochondria. At the same time, its double-layered phospholipid structure allows for free exchange of metabolites and cations on both sides of the mitochondria.^19^ Outer mitochondrial membrane can simultaneously contact and interact with several other cell structures, such as the endoplasmic reticulum, ribosome, and nucleus.^20^^,^^21^ Mitochondrial outer membrane permeability has an important relationship with mitochondrial cell death, which is regulated by the B-cell lymphoma 2 (Bcl-2) protein family.^22^ Through mitochondrial outer membrane permeability, mitochondria release a series of pro-apoptotic proteins into the cytoplasm, including cytochrome C (CytC), AIF, Smac/Diablo, serine protease HtrA2/Omi, and endonuclease G,^23^ which participate in the key process of apoptotic cell death.^24^ Cancer cells typically develop resistance to mitochondrial outer membrane permeability induction, thereby avoiding cell death.^25^ The intermembrane space of mitochondria, located between the outer mitochondrial membrane and inner mitochondrial membrane, is full of amorphous liquid. The inner mitochondrial membrane is located inside the outer mitochondrial membrane, and part of the inner mitochondrial membrane folds into the mitochondrial matrix to form mitochondrial cristae. At the base of the cristae, the inner membrane shrinks to form cristae junctions.^18^ The process of remodeling, fusion, and fusion of mitochondrial cristae, collectively known as mitochondrial dynamics, can alter mitochondrial function to promote cell survival. Also, mitochondrial cristae provide an important site for OXPHOS.^26^ The mitochondrial matrix is the part of mitochondria wrapped by the inner mitochondrial membrane, which contains mitochondrial ribosomes and mtDNA. The mtDNA is a closed-loop double-stranded structure with a length of 16,569 bp.^19^ It encodes 37 genes related to cellular respiration, including 13 mRNAs, 2 rRNAs (12S and 16S), and 22 tRNAs.^27^ A recent research reveals the 14th protein named CYTB-187AA is also encoded by mtDNA.^28^ Different from the other 13 proteins synthesized within mitochondria, CYTB-187AA is synthesized in the cytoplasm in accordance with the standard genetic code translation, which the researchers called mtDNA-encoded protein arising from cytosolic translation.^28^

## Function of mitochondria

eMitochondria play a key role in cellular respiration. Mitochondria contain all enzymes required for the TCA cycle, including citrate synthase (CS), aconitase 2 (ACO2), isocitric acid dehydrogenase (IDH), α-KG dehydrogenase (α-KGDH) complex, succinyl CoA synthase (SCS), succinate dehydrogenase complex (SDH), fumarate hydratase (FH), and malate dehydrogenase 2 (MDH2).^27^^,^^29^ This cycle generates pyruvate and fatty acids from glycolysis. β-Acetyl coenzyme A produced during oxidation generates water and carbon dioxide as well as reduced nicotinamide adenine dinucleotide (NADH) and reduced flavin adenine dinucleotide (FADH2), which provide electrons to the electron transfer chain (ETC) of the inner mitochondrial membrane.^30^ Mitochondrial OXPHOS contains five enzyme complexes, namely, NADH ubiquinone oxidoreductase (complex I), succinate ubiquinone oxidase (complex II), ubiquinone cytochrome C oxidoreductase (complex III), cytochrome C oxidase (complex IV), and ATP synthase (complex V),^31^ and ETC contains complex I-IV, as well as the electron transport agents (ubiquinone and CytC). In the process of OXPHOS, NADH and FMNH2 are oxidized through the respiratory chain to generate water. At the same time, the free energy released by NADH and FMNH2 is used to couple ADP phosphorylation to generate a large amount of ATP, which provides energy for cells. OXPHOS is achieved by electron transfer along the respiratory chain, in which by-products ROS are produced.^30^ In addition to cellular respiration, mitochondria participate in key physiological processes, including cell signal transduction, the urea cycle, cell apoptosis, regulation of cytoplasmic calcium concentration, and iron–sulfur cluster biogenesis.^32^ Therefore, mitochondrial dysfunction is likely to affect a range of critical cellular functions and lead to various diseases, including cancer.

## Mitochondria and cell death

### Apoptosis

**Figure 1:**
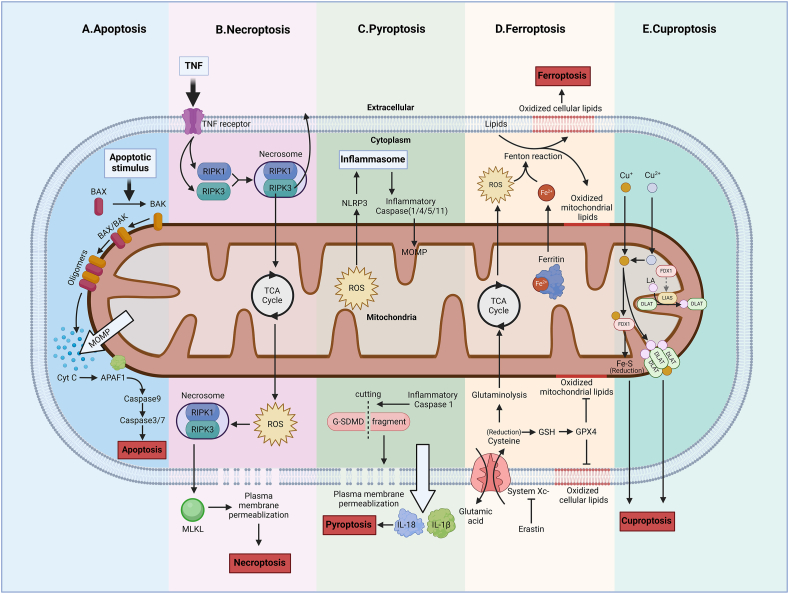
Mitochondria-associated cell death pathways. Mitochondria are involved in apoptosis, necrosis, pyroptosis, and iron death. At present, the research on cell apoptosis is relatively thorough, and the mechanism of mitochondria in it has also been elucidated. Mitochondria are indispensable in the process of cell apoptosis. The research on the other three cell death modes is not as clear as cell apoptosis, and the role of mitochondria in them is not indispensable. **(A)** Apoptosis. The BCL2 protein family drives MOMP to promote mitochondrial CytC release from the intermembrane space, then combines with APAF1, and finally activates caspase to cause apoptosis.^35^, ^36^, ^37^, ^38^ IPAs can inhibit cell apoptosis by inhibiting the activation of caspase-3 and caspase-7.^274^**(B)** Necrosis. RIPK1 and RIPK3 form a tumor to activate MLKL, and make the cell membrane permeable, leading to endocytosis.^41^ Mitochondria can promote phosphorylation of RIPK1 through ROS,^42^, ^43^, ^44^ thereby promoting necrosis. RIPK3 can, in turn, promote the increase of ROS.^37^^,^^44^^,^^45^**(C)** Pyroptosis. Inflammatory caspases cut GSDMD and activate it, making the cell membrane permeable, releasing inflammatory factors, and leading to cell death.^49^^,^^50^ The generation of ROS by mitochondria in response to stress can activate NLRP3 inflammasomes, thereby inducing pyroptosis. At the same time, activated caspase-1 can also initiate mitochondrial apoptosis.^47^^,^^51^**(D)** Ferroptosis, when ferritin in mitochondria chelates with free iron ions in cells. When mitochondrial function is abnormal, iron ion accumulation participates in the Fenton reaction, causing lipid peroxidation, resulting in lipid accumulation and imbalance of intracellular redox balance, leading to cell death.^24^^,^^52^^,^^62^**(E)** Cuproptosis. FDX1 reduces Cu^2+^ to Cu^+^ and facilitates the lipoylation of DLAT. Accumulated Cu^+^ binds to lipoylated DLAT, instigating its aggregation and triggering proteotoxic stress. BAX, BCL2-associated X protein; BAK, BCL2 antagonist/killer; MOMP, mitochondrial outer membrane permeabilization; CytC, cytochrome C; APAF1, apoptotic protease activating factor 1; TNF, tumor necrosis factor; RIPK, receptor-interacting protein kinase; TCA, tricarboxylic acid; ROS, reactive oxygen species; MLKL, mixed lineage kinase domain-like pseudo kinase; NLRP3, nucleotide-binding oligomerization domain-like receptor protein 3; G-SDMD, gasdermin-D; IL-18, interleukin 18; GSH, glutathione; GPX4, glutathione peroxidase 4; FDX1, ferredoxin 1; LA, lipoic acid; DLAT, dihydrolipoamide S-acetyltransferase; LIAS, lipoic acid synthetase.

Mitochondria are involved in energy metabolism in cells and are closely associated with the regulation of cell death. Mitochondria are involved in different cell death modes, including apoptosis, necroptosis, pyroptosis, ferroptosis, and cuproptosis (Fig. 1). Apoptosis is an important mode of pathophysiological cell death and plays a critical role in various homeostatic regulation processes from embryonic development to the human body. Apoptosis is primarily achieved through two signaling pathways: the extrinsic (death receptor) pathway of apoptosis and the intrinsic (mitochondria) pathway of apoptosis.^33^^,^^34^ Mitochondria mediate cell apoptosis through the second pathway,^34^ which requires mitochondrial outer membrane permeability to promote the release of pro-apoptotic factors from the intermembrane space of mitochondria.^35^ Mitochondrial outer membrane permeability is driven by the effector proteomic members of the Bcl-2 protein family, mainly BCL2-associated X (BAX) and BCL2 antagonist/killer (BAK).^36^ BAK is located in the outer mitochondrial membrane, while BAX is generally present in the cytoplasm. When apoptotic signals, such as hypoxia, loss of cell growth factors, activation of oncogenes, and DNA damage, are received, BAX is repositioned on the outer mitochondrial membrane, forming oligomers with BAK, forming pores on the outer membrane of mitochondria, and increasing membrane permeability.^36^, ^37^, ^38^ When both BAX and BAK are absent, cells develop resistance to mitochondrial apoptosis.^33^ Apoptosis-promoting factors (CytC, AIF, ENDOG, *etc*.) in the mitochondrial intermembrane space are released into the cytoplasm. CytC and apoptotic protease activating factor 1 (APAF1) form an apoptosome, activate pro-caspase-9 to form caspase-9 holoenzyme, further activate caspase-3 and caspase-7, then start the caspase cascade reaction, act on various substrates in the cell, and eventually lead to apoptosis.^37^^,^^39^ Mitochondrial outer membrane permeability can also play a role in inducing pro-inflammatory signaling during apoptosis,^33^ and permeabilized mitochondria trigger inflammation through the release of mitochondria-derived damage-associated molecular patterns.^40^ In addition to this, there may be other pathways and roles of mitochondrial outer membrane permeability in apoptosis, and the elucidation of these pathways and the crosstalk and relationships between them may be an important direction for future research.

### Necroptosis

Necroptosis is a kind of regulated caspase-independent cell death, which is often associated with ischemic injury and inflammation. It can be induced by various stimuli such as tumor necrosis factor, viral infection, and Toll receptor signaling. It activates receptor-interacting protein kinase 1 (RIPK1) and RIPK3, forming a necrosome.^41^ RIPK3 activates mixed lineage kinase domain-like pseudo kinase (MLKL), causing cell membrane permeability, leading to necroptosis.^41^ Mitochondria can promote phosphorylation of RIPK1 through ROS,^42^, ^43^, ^44^ thereby promoting necrosis. RIPK3 can, in turn, promote the increase in ROS.^37^^,^^44^^,^^45^ Although the role mitochondria play in necrosis remains unclear, it has been reported that mitochondrial dysfunction can heighten the propensity for cell necrosis.^46^

### Pyroptosis

Pyroptosis is an inflammatory-regulated cell death that can be induced by inflammatory caspases (mainly caspase-1, caspase-4, caspase-5, and caspase-11), which are activated by inflammasomes (including the nucleotide-binding domain leucine-rich repeat (NLR) family NLRP1, NLRP3, NLRC4, and HIN200 family AIM2).^47^ The important participant in pyroptosis is the gasdermin protein (GSDMD), which is activated by caspase cleavage.^48^ The cleaved fragments make holes in the cell membrane, leading to membrane permeability^49^ and the release of various inflammatory factors, such as interleukin-1beta (IL-1β) and IL-18, leading to cell death.^50^ The generation of ROS by mitochondria in response to stress can activate NLRP3 inflammasomes, thereby inducing pyroptosis. At the same time, activated caspase-1 can initiate mitochondrial apoptosis.^47^^,^^51^

### Ferroptosis

Ferroptosis is another kind of regulated cell death, which is a novel type of iron-ion-dependent cell regulatory death induced by lipid peroxides.^52^ The morphology of ferroptosis is characterized by a decrease in mitochondrial volume, a decrease in the order of appearance of mitochondrial cristae, and an increase in the density of mitochondrial membranes in the electron microscope.^53^ Ferroptosis processes are associated with glutathione (GSH) depletion, selenoprotein glutathione peroxidase 4 (GPX4) inactivation, and increased lipid peroxidation.^54^^,^^55^ The peroxidation of lipids requires the Fenton reaction, and as iron ions are necessary for the Fenton reaction, they are an indispensable part of ferroptosis.^56^ Under normal circumstances, peroxidized lipids are reduced and inactivated by glutathione peroxidase 4 (GPX4). GSH is a cofactor for GPX4, and GSH requires cysteine synthesis.^57^^,^^58^ The transport of cysteine into the cytoplasm is driven by System x_c_^−^ transport of glutamic acid out of the cytoplasm.^59^^,^^60^ This process can be inhibited by a small-molecule inhibitor, erastin, leading to lipid accumulation of peroxidation, imbalance of intracellular redox, and cell death.^24^ Iron pools are present in mitochondria, and these iron ions are mainly used for the synthesis of cofactors such as haemoglobin.^61^ The connection between mitochondria and ferroptosis is reflected in two aspects: the ferritin in mitochondria chelates with free iron ions in cells, reducing iron-dependent lipid peroxidation caused by the Fenton reaction.^57^ When mitochondrial function is abnormal, accumulation of excess iron ions in cells produces iron toxicity, and excess iron ions participate in the Fenton reaction, leading to lipid peroxidation of the mitochondrial membrane.^52^^,^^62^^,^^63^ On the other hand, the decrease in cysteine leads to an increase in the decomposition of glutamine. Its decomposition products, as substrates, can enhance the TCA cycle, leading to an increase in ROS production and promoting lipid peroxidation.^24^

### Cuproptosis

The recent findings of Tsvetkov et al have established a significant link between a unique form of cell death, termed “cuproptosis”, and mitochondrial functions.^64^ They have uncovered that an abundance of copper ions in the mitochondria can instigate the oligomerization of lipoylated drolipoamide S-acetyltransferase (DLAT) proteins and the reduction of Fe–S cluster proteins (Fig. 1). This process induces the proteotoxic stress, consequently promoting cell death. While Tsvetkov et al have identified key regulators of cuproptosis, such as ferredoxin 1 (FDX1), lipoic acid synthase (LIAS), lipoyl transferase 1 (LIPT1), DLAT, dihydrolipoamide dehydrogenase (DLD), pyruvate dehydrogenase E1 subunit alpha 1 (PDHA1), pyruvate dehydrogenase E1 subunit beta (PDHB), metal-regulatory transcription factor-1 (MTF1), glutaminase (GLS), and cyclin-dependent kinase inhibitor 2A (CDKN2A), through robust methodologies involving CRISPR-screen screening and knockout validation,^64^ the precise molecular mechanism underlying cuproptosis remains elusive. There is a pressing need for additional research to unravel this mechanism further, providing a clearer understanding of this complex process.^65^^,^^66^

As underscored above, mitochondria serve a crucial regulatory function in diverse modalities of cellular death. Unraveling the molecular mechanisms through which mitochondria orchestrate cell death is paramount for a comprehensive understanding of the genesis and treatment of diseases associated with mitochondrial dysfunction. Moreover, despite the varied mechanisms through which mitochondria regulate different forms of cell death, commonalities exist in the form of shared molecules or pathways involved in these processes, such as ROS and the TCA cycle. This implies that mitochondrial-regulated cell death is not a singular pathway of demise. In future investigations of mitochondrial-related diseases, a holistic approach that considers multiple death modalities may prove advantageous, enabling a full elucidation of the disease pathogenesis and facilitating the development of effective diagnostic and therapeutic strategies.

## Mitochondria and tumorigenesis and tumor progression

### mtDNA mutations and tumors

**Figure 2:**
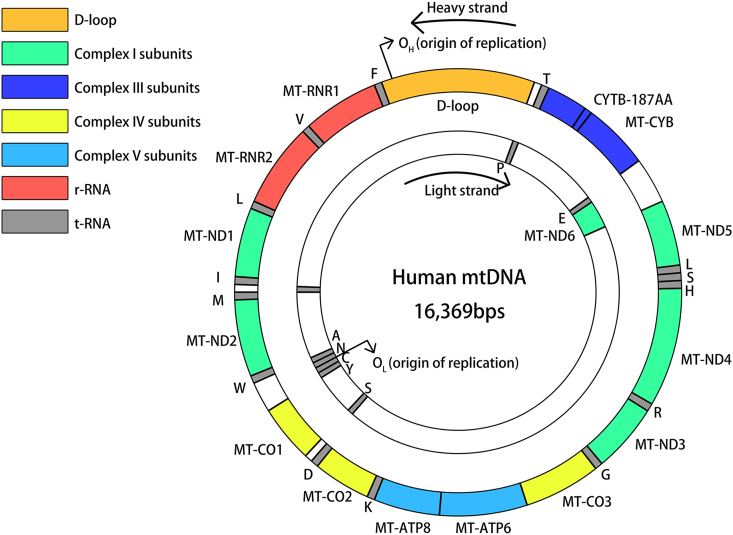
Structure of mtDNA and the location of the RNA it encodes. mtDNA is a double-stranded ring with a heavy strand on the outer ring and a light strand on the inner ring. The length of human mtDNA is 16569 bps.^19^ O_H_ and O_L_ are the replication starting points for the heavy strand and light strand, respectively. mtDNA encodes 37 genes, including 14 polypeptides, 2 mitochondrial ribosomal RNAs, and 22 tRNAs necessary for the OXPHOS system.^27^^,^^28^ The D-loop, OXPHOS complex subunits coding regions, rRNA, and tRNA coding regions are represented in different colors in the diagram. Except for 8 tRNAs and MT-ED6 on the light strand, all others are on the heavy strand.^7^ MT-CYB, mitochondrial cytochrome B subunit; MT–ND, mitochondrial NADH dehydrogenase subunit; MT–CO, mitochondrial cytochrome C subunit; MT-ATP, mitochondrial ATP synthase subunit; MT-RNR, mitochondrially encoded 12S rRNA.

mtDNA is a closed-loop double-stranded DNA molecule containing 16,569 base pairs (Fig. 2). The outer ring of mtDNA contains more guanine, which is called a heavy chain (H chain), whereas the inner ring contains more cytosine, which is called a light chain (L chain).^67^ mtDNA does not bind to histones. It encodes 37 genes, including 14 polypeptides, 2 mitochondrial ribosomal RNAs, and 22 tRNAs necessary for the OXPHOS system. Proteins required for mtDNA replication, transcription, and RNA processing and translation are encoded by the nucleus.^68^ mtDNA follows strict maternal inheritance, with a single mitochondrion containing several copies of the mitochondrial genome, while a cell contains hundreds to thousands of copies. The copy number varies depending on different cell types and primarily depends on the cell's energy requirements.^69^

### Mutation of mtDNA and tumor development and progression

Unlike nuclear DNA, there are no introns in mtRNA, and there are no or few non-coding bases between genes. Compared with nuclear DNA, mtDNA has a higher mutation rate.^70^^,^^71^ The transcription and translation of mtRNA are carried out in mitochondria, and in most cases, there is no stop codon.^72^ The heterogeneity of mtDNA is mainly reflected in the D-loop and some coding regions. The D-loop is the only major non-coding region in mtDNA molecules. There are three hypervariable regions (HVRs) in mtDNA, and the D-loop contains two of them, namely, HVR-I and HVR-II.^73^ The replication of mtDNA begins with the D-loop, which also contains the transcriptional control region of mtDNA. Therefore, mutations in the D-loop may affect mtDNA CN.

Compared with nuclear DNA, mtDNA lacks a repair system for DNA damage, and is close to ROS at the spatial level. Therefore, its mutation rate is high. It is reported that nearly 200 different mutations have occurred in human mtDNA.^74^ For example, in ovarian cancer cells, the mutation rate of mtDNA is as high as 60%.^75^ Mutations in mtDNA are believed to have two effects on tumors: as an “inducer” promoting tumor development and as an “adapter” promoting tumor progression.^7^ mtDNA mutation results, such as anti-apoptosis and epithelial-mesenchymal transformation, may promote the development and development of tumors.^76^ Mutations and damages in mtDNA can lead to damage to the OXPHOS system and an increase in ROS production, which may in turn accelerate the occurrence of DNA mutations.^77^ The increase in ROS generation can lead to oxidative damage to lipids and proteins as well as DNA mutations. Mutations in mtDNA can also alter mitochondrial metabolism to affect cancer occurrence and development. Likewise, changes in mitochondrial metabolism can also affect the epigenetics of mtDNA.^7^

The abundance of mtDNA heterogeneity in the RNA-coding region of the mitochondrial genome was demonstrated to be significantly higher in tumor cells compared with normal cells, and the missense mutations, nonsense mutations, deletions, and insertions in mtDNA were noted to be significantly more frequent in tumor cells than in normal cells.^78^ mtDNA encodes four of the five enzyme complexes in the mitochondrial respiratory chain (except complex II; Fig. 2). NADH dehydrogenase is a component of complex I and is expressed by seven mtDNA genes, ND1–6 and ND4L. Mutations in mtDNA have mostly been reported for complex I, which may alter the OXPHOS process and ROS production in cancer cells. One study found enhanced glycolysis and decreased mitochondrial respiration in cancer cells by mtDNA base editing in a mouse model of melanoma, resulting in a truncating mutation in ND5.^79^ Cytochrome B, a component of complex III, is encoded by the CYB gene in mtDNA, and MT-CYB mutations have been demonstrated to increase ROS production and induce nuclear factor kappa B (NF-kB) signaling-mediated tumor growth in xenograft and human bladder cancer models.^20^ Cytochrome C oxidase, a component of complex IV, is encoded by three genes, CO1–3, in mtDNA. Mutations in CO1 are the most common mtDNA mutations in breast, cervical, and bladder cancers.^80^^,^^81^ In one study, mtDNA mutations were found in 73.9% of breast cancers, and CO1 showed the highest number of mutations.^82^ In colorectal cancer, mutations in CO1 may reduce the efficiency of the mitochondrial respiratory chain. ATP synthase, a component of complex V, is expressed by two genes in mtDNA, ATP6 and ATP8. Mutations in complex V are associated with apoptosis resistance in cancer cells, performing the last step of the OXPHOS process and participating in the formation of an osmotic capacity transition pore for calcium efflux and apoptosis. Mutations in ATP6 and ATP8 have been found in cancers, including thyroid cancer,^83^ breast cancer,^84^ pancreatic cancer,^85^ and osteosarcoma.^86^ In addition, mutations in the non-coding region, especially in the D-loop region, have been associated with various tumors, including myeloid leukemia,^87^ tongue squamous cell carcinoma,^88^ and hepatocellular carcinoma.^89^ As previously noted, the majority of these tumor-associated mtDNA mutations impede the full functionality of the electron transport chain. This impairment diminishes the efficiency of OXPHOS, concurrently escalating the generation of mitochondrial-derived ROS. This implies that ROS-related, tumor-driving mechanisms hold significant importance in future research on tumors harboring mtDNA mutations.

### Copy number of mtDNA and cancer prediction and prognosis

The decrease in mtDNA CN is associated with point mutations located near the start of D-loop replication and damage occurring in mitochondrial organisms. Since the D-loop region controls mtDNA replication and transcription, mutations in this region result in abnormal mtDNA transcription and translation and a decrease in mtDNA CN.^90^ This decrease leads to a decrease in mitochondrial transcription, down-regulation of OXPHOS-related proteins, and a decrease in mitochondrial respiratory function, and may also lead to impaired processes such as mitochondrial fusion.^8^ mtDNA CN is closely related to tumors and is a good tool for early diagnosis and prognosis of tumors, and its determination can be obtained quantitatively in blood.^8^ mtDNA CN can be quantified more accurately by digital PCR and high-throughput sequencing.^91^ However, the correlation between mtDNA CN and tumors varies widely, with conflicts such as positive and negative correlations, depending on the type of cancer^92^^,^^93^ (Table 1).

**Table 1:** Relationship between the outcomes of different types of cancer and changes in mtDNA copy number.

Cancer type	Changes of mtDNA copy number	Outcomes	References
Colorectal cancer	Increased	Poor prognosis	95
	Decreased	Lower risk of death	96
	Increased or decreased	Elevated risk of morbidity	98
Gastric cancer	Increased or decreased	Elevated risk of morbidity	98
Esophagus cancer	Increased or decreased	Elevated risk of morbidity	98
Pheochromocytoma	Decreased	Decrease in the number of mitochondria	99
Paraganglioma	Decreased	Decrease in the number of mitochondria	99
Glioblastoma	Decreased	Compared to normal tissue	100
	Decreased	Increase in survival rate	101
	Increased	Increased overall survival in young adults	102
Pancreatic ductal adenocarcinoma	Increased	Decreased risk of morbidity	103
Breast cancer	Decreased	Compared with non-patients	24
	Increased	Compared with normal tissue	24
	Decreased	Good prognosis	18
Prostate cancer	Increased	Increased biochemical recurrence	106
Ovarian tumor	Increased	Increased deterioration of the tumor	107

The decrease in mtDNA CN in hepatocellular carcinoma correlates with tumor size, degree of cirrhosis, five-year survival, and mitochondrial copy number in peripheral blood leukocytes as a possible predictor of hepatocellular carcinogenesis.^77^ A U-shaped association between peripheral blood mtDNA CN and the risk of hepatocellular carcinoma was found in one study; that is, high or low levels of peripheral blood mtDNA CN could increase the risk of hepatocellular carcinoma.^94^ In one study, an increase in mtDNA CN was found in colorectal cancer tissue and was associated with poor patient prognosis.^95^ Another study demonstrated that reduced mtDNA CN at the time of diagnosis is associated with a lower risk of colorectal cancer-associated mortality.^96^ It has also been reported that a functional pool of Src homology 2 domain-containing F (SHF) in mitochondria can increase mtDNA CN and thus reduce the sensitivity of colorectal cancer to radiation. Therefore, the inhibition of SHF may be a potential target to improve the efficacy of radiotherapy for colorectal cancer.^97^ However, in another study, a significant U-shaped association between mtDNA CN and the risk of gastrointestinal cancers was found in three types of gastrointestinal cancers: intestinal, gastric, and esophageal cancers.^98^ In one study, a decrease in mtDNA CN was found in more than 87% of pheochromocytomas and paragangliomas, accompanied by a decrease in pro-mitochondriogenic regulators, resulting in a decrease in the number of mitochondria.^99^ In glioblastoma, mtDNA CN was found to be significantly lower in brain tissue than in normal tissue, and the same results were shown in peripheral blood mtDNA CN.^100^ A study found that lower mtDNA CN in glioblastoma was associated with survival, and mtDNA CN was higher in patients treated with radiation therapy than in newly diagnosed patients.^101^ However, in another study, higher mtDNA CN was found to be significantly associated with overall survival in young adults with glioblastoma.^102^ In a case–control study of ductal adenocarcinoma of the pancreas, an increase in mtDNA CN was found to be associated with a decrease in the incidence of ductal adenocarcinoma of the pancreas.^103^ In breast cancer, a study found significantly less mtDNA CN in blood samples from patients compared with those from non-patients but significantly higher mtDNA CN in tumor tissue compared with that in non-tumor tissue in patients.^24^ Furthermore, in one study, a reduction in mtDNA CN in breast cancer patients was found to be a possible sign of a good prognosis.^104^ Correspondingly, it has been demonstrated experimentally that a reduction in mtDNA CN leads to a reduction in the proliferation of triple-negative breast cancer cells.^105^ A prostate cancer study demonstrated an increase in blood leukocyte mtDNA CN associated with increased biochemical recurrence in patients with prostate cancer.^106^ In patients with serous epithelial ovarian tumors, mtDNA CN was significantly elevated and increased according to the degree of tumor progression.^107^ In a recent meta-analysis, mtDNA CN was found to be associated with lip, oral, and testicular cancers, but the exact correlation has yet to be verified due to the small number of included patients.^108^ mtDNA has good potential in cancer prediction and prognosis, but the specific correlation for each cancer needs to be further investigated. The correlation between mtDNA CN and cancers varies among studies, and the magnitude of the correlation may not be sufficient to support the diagnosis, so further studies are required to investigate this correlation.

### Epigenetics in mtDNA and carcinogenic effects

The epigenetic mechanism of mtDNA is also related to carcinogenic effects. Epigenetic modifications of mtDNA include methylation and hydroxymethylation,^109^ and methylation of mtDNA has been proven to contribute to tumor development.^9^ mtDNA methylation is carried out by mtDNA methyltransferase, which transfers methyl groups from the donor S-adenosyl-l-methionine to the C5 position of cytosine, forming 5-methylcytosine.^110^^,^^111^ This process occurs in the D-loop region of mtDNA and follows maternal inheritance.^19^ In cancer, eight abnormal methylation sites are tightly dysregulated in the D-loop region.^112^ In a study, the methylation rate of the D-loop region was significantly reduced in 65 colorectal cancer tissues, with clinical pathology stages III and IV significantly lower than stages I and II, and an increase in mtDNA CN compared with normal tissues.^113^ Further research has demonstrated that demethylation of certain specific sites (CpG sites at positions 4 and 6/7) in the D-loop can lead to an increase in mtDNA CN in colorectal cancer.^114^ A study targeting glioblastoma and osteosarcoma also showed a decrease in methylation levels at multiple sites in mtDNA observed from early to late stages of cancer.^115^ These studies indicate a negative correlation between methylation levels in the D-loop region and mtDNA CN and tumor progression, possibly due to an increase in 5-methylcytosine in the D-loop, limiting mtDNA replication. Empirical evidence has proven that mtDNA methylation is also regulated by feedback from mtDNA CN.^9^ As the findings of the current research on the role of mtDNA epigenetics in cancer are ambiguous, more accurate methods are required to determine the sites of mtDNA methylation and conduct systematic comparative studies with normal tissues to elucidate the function, mechanism, and clinical significance of mtDNA methylation.

### Mitochondrial metabolism and tumors

One of the major metabolic characteristics of tumor cells is metabolic reprogramming to maintain their survival under conditions such as gene change, low nutrient utilization, and hypoxia.^11^ Metabolic reprogramming of tumor cells is primarily manifested as hyperactive glycolysis and impaired aerobic metabolism, which is called “aerobic glycolysis”, also known as the Warburg effect.^116^ However, the metabolic abnormalities in tumor cells are much more complex than the above processes. Recent studies have shown that there is still mitochondrial aerobic metabolism in tumor cells. It has also been found that in cancer cells, cell proliferation and survival can be maintained through metabolic changes in other substances such as lipids, amino acids, nucleic acids, and glutamine.^117^ The primary purpose of anti-tumor therapy targeting tumor metabolic enzymes is to affect the rate of tumor cell synthesis and metabolism, thereby inhibiting the proliferation of tumor cells.

### Glucose metabolism and cancer metabolism changes

Under sufficient oxygen conditions, normal cells in the human body usually undergo aerobic oxidation of glucose for energy supply. After glycolysis, one molecule of glucose is broken down into two molecules of pyruvate and enters the TCA cycle in the form of acetyl-CoA in mitochondria.^118^ The generated NADH and FADH provide electrons for ETC, ultimately producing ATP.^119^ However, in the last century, German physiologist Otto Warburg observed that most tumor cells consume a large amount of glucose but do not have sufficient production capacity. They still ferment glucose to produce lactic acid under sufficient oxygen conditions.^4^ In subsequent studies, Warburg proposed that the effect was ascribable to the impaired respiration of cancer cellular respiration.^120^ However, this was eventually proven to be an erroneous concept. The relative increase of glycolysis of cancer cells under aerobic conditions was caused by the impaired regulation of glycolysis, a phenomenon described as mitochondrial overload.^121^^,^^122^ The debunking of this erroneous theory has taught us that, in addition to their role as bystanders in cancer, mitochondria also play a key role in the metabolism of cancer cells.

**Figure 3:**
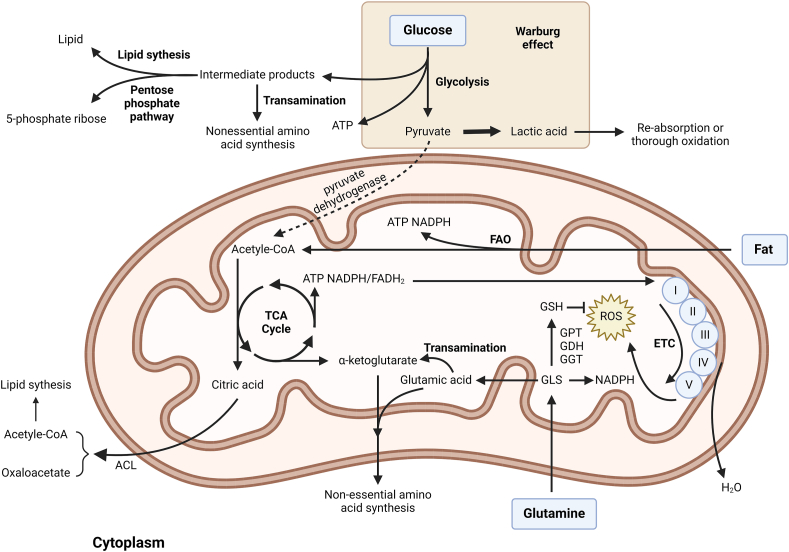
Metabolism of glucose, fat, and glutamine in mitochondria. Glucose is oxidized to pyruvate through glycolysis in the cytoplasm and enters the TCA cycle in mitochondria in the form of acetyl-CoA.^119^ The inhibition of aerobic respiration of mitochondria in tumor cells creates the illusion that cells still undergo glycolysis under aerobic conditions.^24^ The metabolites in glycolysis can be used for the biosynthesis of various other substances.^125^ The intermediate products of the TCA cycle can also be used for other biosynthesis.^126^ FAO serves as an energy source in certain tumor cells,^30^ while also providing NADPH,^133^ used to maintain cell growth and proliferation, as well as respond to oxidative stress.^134^^,^^135^ In other types of cancer cells, the synthesis pathway of fat is greatly enhanced, and most of the fat produced is mainly used for biofilm construction.^128^ Amino acid metabolism also plays an important role in tumor cells. In addition to serving as a substrate for protein synthesis, amino acids can also drive energy and nucleotide generation, as well as maintain cellular redox homeostasis.^34^ Increased metabolism of glutamine is a key feature in many cancer cells.^129^ Glutamine can participate in energy metabolism, promote cell oxidation, and provide NADPH.^145^^,^^146^^,^^148^^,^^149^^,^^151^ ATP, adenosine triphosphate; NADPH, reduced nicotinamide adenine dinucleotide phosphate; FAO, fatty acid oxidation; TCA, tricarboxylic acid; FADH2, reduced flavine adenine dinucleotide; ACL, ATP citrate lyase synthase; CoA, coenzyme A; ETC, electron transfer chain; GLS, glutaminase; GSH, glutathione; GPT, glutamic-pyruvic transaminase; GDH, glutamic dehydrogenase; GGT, gamma-glutamyltransferase.

The Warburg effect of tumor cells causes cells to produce a large amount of lactic acid, resulting in an increase in lactic acid levels and acidic pH in the tumor microenvironment.^123^ A large amount of lactic acid in the microenvironment can produce promoting effects on tumor cells, including inhibiting immune cell activity and promoting epithelial-mesenchymal transformation.^124^ Although cancer cells produce a large amount of lactic acid through glycolysis by consuming a large amount of glucose, not all the glucose consumed is used to provide energy. In addition, it is important to utilize the metabolites of glycolysis for the biosynthesis of various other substances^125^ (Fig. 3). For example, 3-phosphoglycerol can be used as a precursor to synthesize lipids. 6-phosphate glucose, 6-phosphate fructose, and 3-phosphoglyceraldehyde can be metabolized to 5-phosphate ribose through the pentose phosphate pathway and used for *de novo* synthesis of nucleic acids. 3-phosphate glycerate, phosphoenolpyruvate, and pyruvate can be used to synthesize nonessential amino acids through transamination. The metabolic end-product lactate can be reabsorbed and completely oxidized by stromal cells or tumor cells, which largely inhibits the utilization of pyruvate by mitochondria.^125^ In most cancer cells, the pyruvate kinase M1, which catalyzes the final step of glycolysis to produce pyruvate, is not expressed. Instead, the isoenzyme pyruvate kinase muscle isoenzyme 2 (PKM2) with lower activity is up-regulated, which inhibits the production of pyruvate and accumulates a large amount of glycolytic products upstream for synthesis metabolism.^126^

In addition to glycolysis, the TCA cycle of tumor cells is disrupted, allowing their intermediate products to be used for other biosynthesis purposes (Fig. 3). Glucose undergoes the TCA cycle and is metabolized into citric acid, which is transported from mitochondria to cytoplasm and catalyzed by ATP citrate lyase synthase (ACL) to produce oxaloacetate and acetyl CoA, which are precursors for lipid synthesis.^127^ Up-regulation of ACL expression in various tumor cells promotes the production of citric acid in the TCA cycle for lipid synthesis.^128^ Furthermore, other metabolic intermediates of TCA, such as oxaloacetic acid and α-ketoglutaric acid, can be used for the synthesis of nonessential amino acids.^129^ This “shrinking” TCA cycle has been shown to be a downsized mitochondrial activity stimulated by the Warburg effect.^130^

### Lipid metabolism and cancer oxidative stress

The fatty acid β-oxidation (FAO) pathway of different cancer cells is related to the type of cancer cells. Studies have found through 13C NMR spectroscopy analysis that less than 50% of acetyl-CoA in glioblastoma comes from blood-derived glucose, indicating that tumor cells can also utilize other substrates besides glucose.^131^ In certain cells, such as prostate cancer cells, the oxidative metabolism pathway of fatty acids is up-regulated, which mainly relies on the oxidative metabolism of fatty acids to provide energy.^132^ In addition to providing ATP, FAO is the main pathway to provide NADPH^133^ (Fig. 3). NADPH plays a key role in cancer cells. On the one hand, NADPH is a coenzyme that synthesizes metabolic enzymes and is crucial for maintaining cell growth and proliferation. On the other hand, NADPH can provide redox ability to resist oxidative stress, which has been proven to be crucial for the survival of cancer cells under metabolic stress conditions.^134^ For example, it has been reported that the inhibition of FAO in glioblastoma results in a decrease in NADPH levels, an increase in intracellular ROS levels, and cell death.^135^

On the contrary, in some other types of cancer cells, the fat synthesis pathway is greatly enhanced, and most of the fat produced is mainly used for biofilm construction.^128^ The main metabolic enzyme involved in this process is citrate lyase, which catalyzes the conversion of citric acid generated by TCA into acetyl-CoA as a precursor for fat synthesis.^127^ Inhibiting the activity of citrate lyase can limit tumor growth, which has been confirmed via multiple experimental models.^136^ The different dependencies of cancer cells and normal cells on FAO can provide new ideas for targeted cancer cell therapy, which has almost no side effects on normal cells.

### Amino acid metabolism/antioxidation and tumor death

Amino acid metabolism also plays a critical role in tumor cells. Besides serving as a substrate for protein synthesis, amino acids can drive energy and nucleotide generation as well as maintain cellular redox homeostasis^6^ (Fig. 3). It has been found that many types of cancer cells are unable to synthesize some nonessential amino acids, but in normal cells, they can be synthesized directly through transamination, which is caused by mutations or silencing of enzymes involved in the synthesis of these amino acids.^137^ For example, arginine succinate synthase is not expressed in some melanomas, prostaglandin adenoma, bladder cancer, *etc*., and these malignant cells therefore have a unique ability to synthesize arginine *de novo*.^138^^,^^139^ Aspartate and glutamic acid also have deficiencies in their original synthesis pathway-related synthetases and the emergence of special synthesis pathways in some tumor cells.^137^^,^^140^^,^^141^ Inhibition of the specific synthesis pathways of these amino acids in tumor cells may serve as a potential targeted therapy method.

Increased metabolism of glutamine is a key feature in many cancer cells.^129^ Glutamine is a substrate for the cyclic oxidation of tricarboxylic acids and an important starting material for the synthesis of amino acids, nucleotides, and GSH^142^ (Fig. 3). Glutamine is converted into glutamic acid catalyzed by GLS, which can produce nonessential amino acids through transamination or be transferred into mitochondria for conversion. α-ketoglutaric acid enters the TCA cycle.^143^^,^^144^ When glucose is deficient, glutamine derivatives, such as fumarate, malate, and citrate, significantly increase, which can drive the non-glucose-dependent TCA cycle.^145^ In many tumor cells, the accelerated consumption of glutamine leads to accelerated growth of tumor cells.^146^ Carcinogenic transcription factor c-Myc can activate the expression and metabolism of glutamine in cancer cells.^147^

In addition to participating in energy metabolism, glutamine plays a pivotal role in promoting cellular antioxidant activity.^148^ Glutamine derivative glutamic acid can be used for the *de novo* synthesis of GSH. GSH is a tripeptide composed of glutamic acid, cysteine, and glycine. Its synthesis is primarily catalyzed by glutamyl transferase, glutamate hydroxylase, and glutamate glycosyltransferase, which plays an important antioxidant role in cells.^149^^,^^150^ When the metabolism of glutamine increases, it can enhance the antioxidant capacity of GSH and reduce intracellular ROS levels.^151^ In addition to synthesizing GSH, glutamine is the key to providing NADPH, which is essential for fat synthesis and TCA cycling, and can restore oxidized GSH to its reduced state.^149^ As previously mentioned, GSH is a crucial regulatory factor in ferroptosis.^48^, ^49^, ^50^ Additionally, GSH serves as an important companion to copper ions and can capture these ions and thus inhibit cuproptosis.^54^ This suggests that altering the metabolism of GSH within mitochondria can modulate certain modes of cell death, such as ferroptosis and copper-induced cell death, which may be considered a potential strategy for effective cancer treatment.

## Adaptation processes of mitochondria and tumor drug resistance

Drug resistance is a major obstacle in cancer treatment. The formation mechanism of drug resistance is complex and mainly includes drug transport and uptake disorders, the generation of new drug metabolic pathways, drug activation disorders, changes in target enzymes, increased drug decomposition enzymes, production repair mechanisms, and increased drug excretion.^152^^,^^153^ Mitochondria, as the center of energy metabolism in cells, are also involved in drug resistance in tumor cells. Therefore, targeting mitochondria to overcome drug resistance has attracted increasing attention. The various adaptation processes of mitochondria, including mitochondrial fusion and division, mitochondrial metabolism, and mitochondrial autophagy, have been proven to be potential targets for overcoming drug resistance.^3^^,^^154^^,^^155^

### Mitochondrial biogenesis and tumor drug resistance

**Figure 4:**
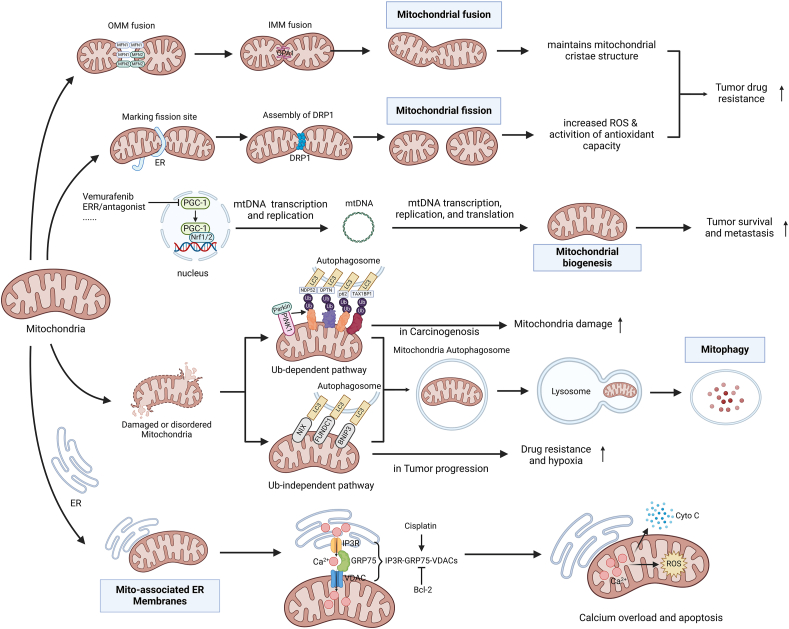
Mitochondrial dynamics and tumor drug resistance. In mitochondrial fusion, MFN1/2 participates in OMM fusion, and then OPA1 mediates IMM fusion and finally completes the mitochondrial fusion process.^3^^,^^154^^,^^171^ In mitochondrial fission, ER first marks the fission site, and then DRP1 assembles and mediates mitochondrial fission here.^3^^,^^154^^,^^171^ Mitochondria fusion and fission can, respectively, maintain mitochondrial cristae structure and increase ROS, eventually leading to increased drug resistance in tumors. In the nucleus, PGC-1 interacts with Nrf1/2, resulting in mtDNA transcription, replication, and translation,^156^^,^^157^ and vemurafenib, ERR/antagonist, *etc*., can inhibit this process.^275^ Mitophagy is mainly mediated through two pathways: the Ub-dependent pathway and the Ub-independent pathway. The former's proteins on mitochondrial membrane are ubiquitinated by PINK1/Parkin and delivered to autophagosome membrane through connexins NDP52/OPTN/p62/TAX1BP1, while the latter's mitochondrial membrane is directly bound to LC3 by NIX/FUNDC1/BNIP3.^162^^,^^163^ Mitochondria are wrapped by an autophagosome, and then fused with a lysosome to finally complete mitophagy.^162^^,^^163^ Oroxylin A, HCQ, Lys05, PIK-III, Gossypol, Phenoformin, *etc*., can inhibit this process.^167^^,^^168^^,^^276^ Mito-associated ER membranes refer to specialized regions where the membranes of the ER and mitochondria are closely apposed or in direct contact.^175^, ^176^, ^177^ IP3R on ER membrane and VDACs on mitochondrial membrane form IP3R-GRP75-VDACs complex via GRP75 connection, which can mediate the transfer of Ca^2+^ from ER to mitochondria.^178^, ^179^, ^180^^,^^192^ This is promoted by cisplatin and inhibited by Bcl-2.^192^, ^193^, ^194^ The transfer of Ca^2+^ can produce calcium overload in the mitochondria and lead to apoptosis through CytC release or ROS production.^188^^,^^189^ OMM, outer mitochondrial membrane; MFN, mitofusin; IMM, inner mitochondrial membrane; OPA1, optic atrophy 1; ER, endoplasmic reticulum; DRP1, dynamin-related protein 1; ROS, reactive oxygen species; PGC-1, peroxisome proliferator-activated receptor-γ coactivator-1; Nrf1/2, nuclear respiratory factor 1/2; LC3, light chain 3 protein; NDP52, nuclear dot protein 52; OPTN, optineurin; p62, sequestosome-1; TAX1BP1, Tax1-binding protein 1; Ub, ubiquitin; PINK1, PTEN induced putative kinase 1; NIX, NIP3-like protein X; FUNDC1, FUN14 domain-containing 1; BNIP3, BCL2-interacting protein 3; IP3R, inositol 1,4, 5-trisphosphatereceptor; GRP75, glucose-regulated protein 75; VDAC, voltage-dependent anion channel; Bcl-2, B-cell lymphoma-2; CytC, cytochrome C.

Through mitochondrial biogenesis, new mitochondria are generated from existing mitochondria to achieve self-renewal.^156^ Increased mitochondrial mass is often detected in cancer stem cells, reflecting an increase in mitochondrial biogenesis. Cancer stem cells have been shown to be resistant to various commonly used chemotherapy drugs in tumors, indicating a correlation between mitochondrial biogenesis and increased tumor chemotherapy resistance.^10^ Peroxisome proliferator-activated receptor-γ coactivator family (PGC-1α, PGC-1β, and PRC) is involved in mitochondrial biogenesis, where PGC-1α plays a central regulatory role in mitochondrial biogenesis while also inhibiting oxidative stress in tumor cells, which can promote the survival and metastasis of tumor cells^156^^,^^157^ (Fig. 4). In a study, in 5-fluorouracil-resistant colorectal cancer cells, the expression of PGC-1α significantly increased, showing enhanced mitochondrial development and antioxidant enzyme activity, indicating that PGC-1α plays a key role in the drug resistance of colorectal cancer cells.^158^ The first targeted drug for hepatocellular carcinoma, sorafenib, is susceptible to drug resistance, and the mechanism of resistance is associated with a reduction in mitochondrial biogenesis.^134^ It has been shown that up-regulated ubiquilin 1 (UBQLN1) induces degradation of PGC-1 and attenuates mitochondrial biogenesis in resistant cells, and patients with higher UBQLN1 levels have reduced relapse-free survival, suggesting that PGC-1β is associated with resistance of hepatocellular carcinoma cells to sorafenib.^159^ PGC-1 may be an important new target for combating cancer cell resistance.

### Mitochondrial autophagy and tumor drug resistance

Mitochondrial autophagy is a selective macroautophagy in which damaged and dysregulated mitochondria can be degraded, cleared, and recycled through the autophagic pathway to maintain mitochondrial and cellular homeostasis.^160^^,^^161^ Mitochondrial autophagy is mediated primarily through two pathways, the phosphatase and tensin (PTEN)-induced kinase1 (PINK1)/Parkin-mediated ubiquitin pathway and the BCL2-interacting protein 3 (BNIP3), BCL2/adenovirus E1B protein-interacting protein 3-like (BNIP3L)/NIP3-like protein X (NIX), and FUN14 domain-containing 1 (FUNDC1)-mediated hypoxia pathways^162^^,^^163^ (Fig. 4). During carcinogenesis, mitochondrial autophagy can inhibit tumor development by reducing mitochondrial damage, a process in which mitochondrial autophagy can be inhibited by Parkin mutation, while during tumor progression, mitochondrial autophagy is increased by abnormal regulation of BNIP3 to enhance drug resistance and anti-hypoxia ability of tumor cells.^163^ Tumor autophagy plays a different role in the development of different tumors to increase the survival probability of tumor cells.^11^

In tumor cells, dysregulation of mitochondrial autophagy may lead to abnormal mitochondrial function and mitochondrial gene mutations, which in turn promote tumorigenesis, progression, and drug resistance. In a study of breast cancer,^164^ mitochondrial autophagy was enhanced in drug-resistant breast cancer cells overexpressing P-glycoprotein, and the excretion of cytotoxic drug MKT-077 was increased, resulting in drug resistance. Increased mitochondrial autophagy in resistant cells was observed in another study comparing pancreatic cancer cells before and after gemcitabine resistance,^165^ both suggesting a vital role of mitochondrial autophagy in tumor drug resistance. In cisplatin-resistant osteosarcoma and ovarian cancer cells, BNIP3 levels were increased, the rate of mitochondrial autophagy was increased, and silencing or pharmacological inhibition of BNIP3 could re-sensitize these cells to cis-diamminedichloro-platinum, suggesting that BNIP3 inhibition is a potential therapeutic strategy for cisplatin resistance.^166^ Cyclin-dependent kinase 9 (CDK9) inhibitors have been reported to inhibit BNIP3 transcription to block mitochondrial autophagy in hepatocellular carcinoma to overcome drug resistance, and oroxylin A has demonstrated promising therapeutic potential.^167^ The question worth considering is whether inhibition of other pathways mediating mitochondrial autophagy could also be a new target for tumor drug resistance. In chronic granulocytic leukemia, treatment with selective BCR-ABL-targeting tyrosine kinase inhibitors (TKIs) resulted in enhanced mitochondrial autophagy, leading to the survival of therapy-resistant leukemic stem cells and TKI resistance, and mitochondrial autophagy inhibitors, such as HCQ, Lys05, and PIK-III, have been used clinically and have been shown to reverse TKI resistance.^168^ By regulating mitochondrial autophagy, it may become a new strategy for tumor treatment, but there are no clinically specific inhibitors of mitochondrial autophagy currently on the horizon.^169^ Its specific mechanism, effect, and better target of action need to be investigated in depth.

### Mitochondrial fusion–fission and tumor drug resistance

Mitochondrial fusion–fission is the process whereby mitochondria fuse or divide in a cell, also known as mitochondrial dynamics. Fusion is the process by which two or more mitochondria merge to form larger mitochondria, thereby improving mitochondrial DNA integrity, mitochondrial respiration, ATP production, and mitochondrial membrane potential, as well as facilitating communication between mitochondria and host cells.^170^ Fission is the process by which one mitochondrion contract and divide to produce multiple smaller mitochondria, which has been shown to coordinate with mitochondrial autophagy and apoptosis^154^ (Fig. 4). The important proteins regulating the mitochondrial fusion process were noted to be mitofusin 1 (MFN1), mitofusin 2 (MFN2), and optic atrophy 1 (OPA1), while the key proteins involved in the mitochondrial division process were found to be dynamin-related protein 1 (Drp1) and fission protein 1 (FIS1).^3^^,^^154^^,^^171^ Mitochondrial dynamics are important for maintaining mitochondrial homeostasis and are associated with cell division, apoptosis, and autophagy.^12^

Recent studies have revealed a relationship between mitochondrial fusion–fission and tumor drug resistance. Up-regulation of mitochondrial chaperonin CLIPB was found in acute myeloid leukemia cells resistant to venetoclax, which up-regulates mitochondrial fusion through interaction with OPA1 and maintains mitochondrial cristae structure to cause drug resistance, whereas deletion of CLIPB leads to mitochondrial fusion loss of mitochondrial fusion and apoptosis.^172^ In addition, it has been found that mitochondrial fusion induced by other pathways can also lead to drug resistance. For example, leptin can mediate mitochondrial fusion via activation of myeloid cell leukemia 1, leading to gemcitabine resistance and cell survival in gallbladder cancer cells.^173^ In addition, increased Drp1-dependent mitochondrial division was found in metastatic breast cancer cells, leading to increased mitochondrial ROS production and retrograde activation of cellular antioxidant capacity and chemoresistance, and inhibition of DRP1 could restore sensitivity to cisplatin.^174^ Further studies revealed the presence of mitochondrial fission factor (Mff) in hepatocellular carcinoma, and the knockdown of Mff down-regulated the expression of Drp1 and restored the sensitivity of cisplatin-resistant cells, inhibiting cell proliferation, migration, and invasion. It has been shown that Mff regulates mitochondrial Drp1 expression and promotes cisplatin resistance in hepatocellular carcinoma cells.^13^ In future studies, it is necessary to gain insight into the regulatory mode of mitochondrial fusion division and its role in resistance to chemotherapeutic drugs to find more precise targets for drug resistance. However, it is important to note that during the process of drug resistance in cancer cells, there is a concurrent reshaping of cellular homeostasis, which includes the mitochondrial-associated glucose metabolic balance. Therefore, a comprehensive consideration of mitochondrial biogenesis and its related metabolic homeostasis in future research would provide a more holistic understanding of the role of mitochondria in drug resistance, thereby proposing more effective solutions.

### MAMs and tumor drug resistance

Except for the adaptive changes in the mitochondria, dynamic interactions with other organelles are also a phenomenon that could have implications for cancer. A representative is mitochondria-associated endoplasmic reticulum membranes (MAMs), which refers to specialized regions where the membranes of the endoplasmic reticulum and mitochondria are closely apposed or in direct contact.^175^ These contact sites are crucial for cellular functions such as calcium signaling, lipid metabolism, and cellular stress response.^176^ Endoplasmic reticulum is the main calcium storage site within the cell, and MAMs promote the transfer of calcium from the reticulum to the mitochondria, which plays an important role in calcium storage and buffering.^177^ MAMs also help initiate the mitochondrial autophagy pathway by facilitating communication between the endoplasmic reticulum and mitochondria.^178^ Several proteins play important roles in MAMs. Inositol 1,4,5-trisphosphate receptor (IP3R) is a channel protein on the endoplasmic reticulum that regulates calcium transfer from the endoplasmic reticulum to the mitochondria.^179^ Volte-dependention channel (VDAC), located in the outer membrane of the mitochondria, is involved in the exchange of ions and metabolites between the mitochondria and endoplasmic reticulum.^180^ MAMs are key platforms for lipid synthesis, relying on cholesterol and triacyl glyceride synthase at MAMs, where phospholipids are synthesized and distributed to other organelles.^181^^,^^182^ In cancer cells, enhanced lipid synthesis in MAMs favorably supports the lipid requirements of biofilms in rapid proliferation.^181^

Abnormal endoplasmic reticulum–mitochondrial interactions have been shown to contribute to the metabolism of cancer cells and their survival under stress.^183^ By enhancing calcium signaling in MAMs, it can increase mitochondrial calcium concentration in tumor cells, promote the TCA cycle and OXPHOS, and provide energy for rapid tumor cell proliferation.^184^^,^^185^ Calcium ion exchange by up- or down-regulating MAMs can play a pro- or anti-cancer role.^186^ However, excessive calcium ion transport may lead to calcium overload in mitochondria, resulting in alteration of mitochondrial outer membrane permeability, thereby inducing apoptosis.^187^ Several approaches can trigger cancer cell apoptosis by modulating MAM proteins to promote Ca^2+^ transport. Cisplatin is a widely used anti-cancer chemotherapy drug. It has been found that cisplatin promotes Ca^2+^ transport from the endoplasmic reticulum to the mitochondria in ovarian cancer cells, thereby causing cell apoptosis.^188^^,^^189^ Meanwhile, long-term use of cisplatin may lead to the development of drug resistance, which is related to the glucose-regulated protein 75 (GRP75).^190^^,^^191^ GRP75, together with IPR3 and VDAC, forms the IP3R-GRP75-VDAC complex. It has been demonstrated that knockdown of GRP75 increases cisplatin-induced apoptosis in ovarian cancer cells, suggesting a decrease in drug resistance.^192^ Similar results were observed in uterine cancer, where down-regulated GRP75 significantly inhibited tumor growth.^180^ It has also been reported that the development of drug resistance may be related to the overexpression of Bcl-2, an apoptosis-associated protein, blocking MAM-mediated Ca^2+^ transfer.^193^^,^^194^ One study showed that Bcl-2 inhibitors caused an increase in Ca^+^ in mitochondria by interacting with IP3R,^195^ and another study reduced drug resistance in ovarian cancer cells by modulating the MAM-Ca^+^ pathway with the Bcl-2 inhibitor ABT737.^196^ In addition, an endoplasmic reticulum chaperone protein, Sigma-1 receptor (Sig-1 R), is also involved in the regulation of calcium transport in MAMs.^197^^,^^198^ Sig-1 R expression is increased in certain tumor cells, such as breast cancer, and elevated levels are associated with drug resistance and poorer prognosis in cancer cells.^199^ The distance between mitochondria and endoplasmic reticulum at contact sites is likewise a calcium regulation mechanism, and its increased distance protects tumor cells from calcium overload and apoptosis.^183^^,^^187^ These prove that MAMs play a role in anti-drug resistance of cancer cells, but the mechanism of chemotherapy sensitization targeting MAMs and the signaling pathway still need to be further explored.

## Mitochondria-targeted tumor therapy

Targeting mitochondria has become a new idea in tumor therapy, including gene therapy targeting mitochondrial gene mutations, drug therapy targeting mitochondrial autophagy, and tumor therapy using mitochondrial properties such as membrane potential, respiration rate, and energy production pathways. Conventional chemotherapeutic agents, such as adriamycin, ciclosporin, chloramphenicol, and cisplatin, inhibit cancer cells through different mechanisms, including depletion of ATP production, induction of membrane permeation, loss of mitochondrial membrane integrity, inhibition of mitochondrial respiratory pathways, and damage to mtDNA.^13^ As shown in Table 2, chemotherapy drugs that affect mitochondrial metabolism and function have been listed.

**Table 2:** Chemotherapy drugs that affect mitochondrial metabolism and function.

Drug	Targets	Function	References
Aromatic DCA derivatives	PDC/PDK axis	Inhibiting PDK activity, reducing the inhibition of PDC activity, weakening the glycolysis pathway, and affecting mitochondrial metabolic reprogramming	201
DCA-loaded tertiary amines			202
Furan			203
Hemin	BACH1	Inhibition of PDH activity reduces the utilization of glucose in the tricarboxylic acid cycle and negatively regulates the transcription of ETC gene, making tumor tissues, such as breast cancer, sensitive to mitochondrial inhibitors	204,205
Vosaroxin	HIF-1	Significantly reduces HIF-1 levels, accompanied by increased mitochondrial ROS production and reduced ATP, resulting in cytotoxicity to human cervical cancer cells	207
Cardamonin	HIF-1	Inhibits HIF-1 expression by inhibiting the mTOR/p70s6k pathway, induces mitochondrial ROS accumulation, and leads to apoptosis of breast cancer cells	208
LW1564	HIF-1	Inhibits mitochondrial respiration to increase mitochondrial oxygen concentration to stimulate the degradation of HIF-1, thereby inhibiting the growth of various cancer cell lines	209
Mito-CP	Mitochondrial membrane potential	Inhibits tumor proliferation by altering mitochondrial bioenergetic pathways	210, 211, 212, 213, 214
IACS-010759	Complex I	Inhibition of complex I. In oxidative phosphorylation-dependent brain cancer and acute myeloid leukemia, IACS-010759 exhibits strong inhibition of proliferation and induction of apoptosis	216,217
BAY87-2243	Complex I	Induces stimulation of mitochondrial ROS production, leading to oxidative damage and cell death, and produces anti-tumor activity in tumors such as melanoma	220
ME-344	Complex I Complex III	It is a potent inhibitor of complex I activity and inhibits complex III, which significantly affects mitochondrial respiration, causes changes in mitochondrial membrane potential, and inhibits heme oxygenase 1	221,222
Metformin	Complex I	Specific inhibition of complex I, stimulation of ROS production, and activation of AMPK inhibited mitochondrial respiration	224,226,228,229
Phenformin	Complex I	Leading to disruption of mitochondrial metabolic function and strongly inhibiting the proliferation and tumor growth of EGFR-TKI-resistant non-small-cell lung cancer cells	230,231
Petasin	Complex I	Disrupts mitochondrial metabolism, leading to severe impairment of nucleotide synthesis and glycosylation, and is cytotoxic to many types of tumors	232
Gboxin	Complex V	Acts on mitochondrial oxidative phosphorylation complex V and inhibits F0F1 ATP synthase activity to suppress oxidative phosphorylation in tumor cells	233,234
MitoTam	Complex I	Specifically targets mitochondria, inhibits mitochondrial complex I, and disrupts the assembly of the respiratory supercomplex	236,237
Lonidamine	Complex II	Inhibiting mitochondrial bioenergetics, stimulating the formation of reactive oxygen species, oxidizing mitochondrial hydrogen peroxide, and inducing autophagic death	243, 244, 245, 246
Atovaquone	Complex III	The ability to inhibit oxygen consumption and ATP production may exert profound anti-proliferative effects in breast, ovarian, and glioma cancers	247, 248, 249
Mdivi-1	Mitochondrial dynamics-related protein 1	Inhibition of mitochondrial division induces abnormal mitosis in cells	251, 252, 253, 254, 255

## Mitochondria-targeted chemical compounds

The metabolic function of mitochondria is crucial for tumorigenesis and progression, and therefore, mitochondrial metabolic pathways are important targets for inhibiting cancer cell growth (Table 2). There is a pyruvate dehydrogenase complex (PDC)/pyruvate dehydrogenase kinase (PDK) axis in the TCA cycle, that is, PDK-mediated phosphorylation inhibits PDC activity, causing cells to favor ATP production via glycolysis over OXPHOS, which is associated with a variety of pathological processes, including cancer.^200^ In recent years, several new studies have found that aromatic DCA derivatives,^201^ DCA-loaded tertiary amines,^202^ and furan^203^ inhibit PDK and show potential for cancer therapy. BTB and CNC homology 1 (BACH1), a kind of heme-binding transcription factor, which reduces glucose utilization in TCA by inhibiting PDH activity and negatively regulates ETC gene transcription, can alter mitochondrial metabolism by targeting BACH1, making tumor tissues, such as breast cancer, sensitive to mitochondrial inhibitors.^204^^,^^205^ Hemin, a kind of small inhibitor of BACH1, can cause the degradation of BACH1 and can effectively inhibit the metastatic process of cancer cells.^205^

Mitochondrial redox homeostasis and mitochondrial membrane stability are also important targets. Hypoxia-inducible factor 1 (HIF-1), a key regulator of hypoxia metabolism in cancer cells, regulates mitochondrial redox homeostasis by reducing oxidant production and enhancing antioxidant capacity. It has been demonstrated to be a promising target for novel cancer therapeutics.^206^ A number of mitochondrial drugs targeting HIF-1 are available. Vosaroxin significantly reduces HIF-1 levels, accompanied by increased mitochondrial ROS production and reduced ATP, and is cytotoxic to human cervical cancer cells.^207^ Cardamonin inhibits HIF-1 expression via the mammalian target of rapamycin (mTOR)/70 kDa ribosomal protein S6 kinase (p70s6k) pathway and induces mitochondrial ROS accumulation, leading to apoptosis in breast cancer cells.^208^ Disubstituted adamantyl derivative LW1564 inhibits mitochondrial respiration to increase intra-mitochondrial oxygen concentration to stimulate HIF-1 degradation, thereby inhibiting the growth of various cancer cell lines.^209^ Mitochondria-targeted cationic carboxyproxyl nitroxide (Mito-CP) is a mitochondria-targeted drug with superoxide dismutase activity. It has been demonstrated that Mito-CP inhibits tumor proliferation not by altering mitochondrial superoxide activity but by altering mitochondrial bioenergetic pathways,^210^ which mainly involves the alteration of mitochondrial membrane potential.^211^^,^^212^ In several studies, the synergistic effect of Mito-CP and 2-deoxyglucose can effectively inhibit the growth of pancreatic cancer, breast cancer, medullary thyroid cancer, hepatocellular carcinoma, and other tumors.^210^^,^^212^, ^213^, ^214^

Enzyme complexes and other components in OXPHOS are also used as an important chemotherapeutic drug target. OXPHOS appears to be up-regulated in certain cancers. OXPHOS inhibitors can be applied to these types of cancer cells to alleviate therapeutically adverse tumor hypoxia.^215^ Current research on targeted drugs for the enzyme complexes in OXPHOS is focused on complex I, IACS-010759, a clinical-grade small-molecule inhibitor of complex I of ETC. In OXPHOS-dependent brain cancer and acute myeloid leukemia, IACS-010759 exhibited strong inhibition of proliferation and induction of apoptosis.^216^^,^^217^ However, a recent phase I clinical trial in acute myeloid leukemia failed due to its narrow therapeutic index and drug toxicity.^218^^,^^219^ BAY87-2243 is also a selective small-molecule inhibitor of complex I, which induces stimulation of mitochondrial ROS production, leading to oxidative damage and cell death, and produces anti-tumor activity in tumors such as melanoma.^220^ ME-344, a second-generation synthetic isoflavan analogue, is a potent inhibitor of complex 1 activity and inhibits complex III, which significantly affects mitochondrial respiration, causes changes in mitochondrial membrane potential, and inhibits heme oxygenase 1 (HO-1), showing anti-cancer activity.^221^^,^^222^ Metformin is a first-line drug for the treatment of type 2 diabetes mellitus,^223^ and several studies have shown that a treatment regimen containing metformin can significantly reduce the incidence of colorectal and pancreatic cancer,^224^^,^^225^ where it inhibits mitochondrial respiration by specifically inhibiting complex I,^226^ stimulating ROS production, and activating AMP-activated protein kinase (AMPK).^227^ Metformin has also been shown to have a therapeutic effect in non-small-cell lung cancer by inhibiting mitochondrial OXPHOS.^228^^,^^229^ In a study, biguanide phenformin strongly inhibited the proliferation and tumor growth of epidermal growth factor receptor (EGFR)-TKI-resistant non-small-cell lung cancer cells by targeting the mitochondrial OXPHOS complex 1, which led to disruption of mitochondrial metabolic function^230^ and was also effective in hepatocellular carcinoma.^231^ Recent studies have found that petasin, a highly potent inhibitor of complex 1, is over 1700 times more active than metformin or phenformin, and its inhibition of complex 1 disrupts mitochondrial metabolism, leading to severe impairment of nucleotide synthesis and glycosylation. Furthermore, it is cytotoxic to many types of tumors and does not show toxicity to normal cells.^232^ Gboxin, a small molecule that specifically inhibits the growth of glioblastoma cells and various other cancer cell lines, acts on mitochondrial oxidative phosphorylation complex V and inhibits F_0_F_1_ ATP synthase activity to suppress OXPHOS in tumor cells.^233^^,^^234^ Gboxin has also been observed to have inhibitory effects in hepatocellular carcinoma and to have a synergistic effect with metformin in treatment.^235^ MitoTam is a tamoxifen analogue that specifically targets mitochondria, inhibits mitochondrial complex I, and disrupts the assembly of the respiratory supercomplex, effectively killing many types of cancer cells.^236^ MitoTam has been shown to significantly inhibit high HER2-expressing breast cancer cells.^237^ Zuzana Bielcikova and her team^238^ have entered phase I/Ib clinical trials for mitochondria-targeted MitoTam for the treatment of metastatic solid tumors. Coenzyme Q, also known as ubiquinone, is a fat-soluble coenzyme that is widely found in mitochondria. It is a component of the cellular respiratory chain and plays a role in transferring hydrogen and electrons. A ubiquinone derivative, mitoQ, can target mitochondrial phospholipid membranes and produce antioxidant effects that can affect mitochondrial homeostasis, and current studies have demonstrated its role in aging, hepatitis C, chronic kidney disease, *etc*..^239^^,^^240^ Some recent studies have found that the combination of mitoQ with some pre-existing anti-cancer drugs can enhance the anti-cancer effects,^241^^,^^242^ which suggests that our combination of some traditional anti-cancer drugs with drugs targeting mitochondria or affecting mitochondrial homeostasis may produce better anti-cancer effects.

Lonidamine is a tumor-selective complex II inhibitor that inhibits mitochondrial bioenergetics, stimulates ROS formation, oxidizes mitochondrial hydrogen peroxide, inactivates protein kinase B (AKT)/mTOR/p70S6K signaling, and induces autophagic death in lung cancer cells, thereby blocking the development and metastasis of lung tumors.^243^ Furthermore, it has shown excellent anti-cancer ability in combination with other anti-cancer drugs in several studies.^244^, ^245^, ^246^ Atovaquone, a US FDA-approved drug for malaria, is a complex III inhibitor that inhibits oxygen consumption and ATP production^247^ and slows the growth of ovarian cancer in a mouse model.^248^ Findings of a previous study suggest that atovaquone may play a profound anti-proliferative role in breast, ovarian, and glioma cancers.^249^ Other studies have shown that atovaquone can reduce the tumor load and aggressiveness of acute myeloid leukemia.^250^ The mitotic process of mitochondria can also be a drug target: Mitochondrial division inhibitor 1 is a small-molecule drug that specifically inhibits the mitochondrial division process, and its site of action is mitochondrial dynamics-related protein 1,^251^ which induces abnormal mitosis in cells,^251^, ^252^, ^253^ effective in treating a variety of cancers including thyroid, breast, and ovarian cancers.^251^^,^^254^^,^^255^

Currently, the development of chemical drugs specifically targeting mitochondria is rapidly developing and has important applications in cancer therapy. However, tumor specificity and drug resistance may become limiting factors for the application of mitochondria-targeted drugs. Future research should pay more attention to solving these problems.

## Mitochondria-targeted drugs based on nanoparticles and nanomaterials

Nanoparticle- and nanomaterial-based mitochondria-targeted drug research is one of the emerging research directions in recent years. Traditional cancer treatments such as chemotherapy and radiotherapy can produce irreversible damage to healthy cells, while nanotechnology-based drug therapy can achieve efficient targeting and selective release of cancer cells.^256^ Nanotechnology has excellent physical, chemical, and biological properties that enable efficient delivery and targeted release, bypassing barriers to drug delivery.^257^ The modification of nanoparticles with peptides, inducers, or lipophilic cations can increase their mitochondria-targeting effects, and the encapsulation of anti-cancer drugs in nanoparticles can achieve precise mitochondria-targeting therapy, improve drug efficacy, and reduce toxic side effects (Table 3). For instance, nanographene is an important carbon-based nanomaterial that can be modified with a variety of substances to prepare mitochondria-targeted nanomaterials for mitochondria-targeted photodynamic therapy.^258^ Using nano-graphite modified with indole green-loaded polyethylenimine, this nano-targeting system was shown to exhibit highly selective anti-cancer efficiency against osteosarcoma *in vitro* and *in vivo*.^259^ In a study by Ying Zhang,^260^ a multi-targeted graphene nanosystem based on Fe_3_O_4_, folic acid, and triphenylphosphine was prepared to enhance the effectiveness of tumor photodynamic therapy. Moreover, a team from Japan has designed a mitochondrial targeting vector “MITO-Porter”.^261^ This is a nanoparticle loaded with anti-cancer drugs by liposome encapsulation with mitochondrial membrane protein recognition peptide on its surface, which can enter mitochondria through membrane fusion.^262^ There have been numerous studies applying this vector for mitochondria targeting, such as the delivery of adriamycin into mitochondria to kill drug-resistant kidney cancer cells^263^ and the delivery of pi-extended porphyrin-type photosensitizer to optimize cancer photodynamic therapy.^264^ Another new study found that amphiphilic stachydrine–octadecane conjugate combined with triptolide–liposome acted as a targeting molecule and showed inhibition of tumor growth and reduction of tumor volume in a mouse model of pancreatic cancer.^265^ Poly lactic co-glycolic acid (PLGA) is a nanoparticle of poly(lactic acid), which is piggybacked on paclitaxel (PTX) to form PLGA-PTX. Compared with free PTX, encapsulated PTX maintains preferential cytotoxicity against various colorectal cancer cells while effectively protecting healthy cells.^266^ Triphenylphosphonium (TPP) is a cationic lipophilic molecule, whose lipid solubility enables it to cross cellular and mitochondrial membranes, and can play a good mitochondria-targeting role.^257^^,^^267^ Various anti-cancer drug nano-targeting systems have been developed based on TPP. For example, the combination of antineoplastic agent hydroxycamptothecin (HCPT) and TPP to form the resultant TPP-HCPT conjugate has exhibited superior anti-tumor effects in the treatment of breast cancer.^268^ TPP combined with carbon nanodots (CNDs), GSH-responsive disulfide linker (S–S), and camptothecin (CPT) to form the nanodrug TPP-CNDs-S-CPT showed specific tumor therapeutic activity and minimal side effects.^269^ Encapsulation of lonidamine (LND), short-chain TTP glycol succinate (TPP-TPGS, TPS), and a tumor-targeting long-chain molecule, DSPE-S-S-PEG2000-R6GD (DSSR), in poly(lactic-co-glycolic acid) (PLGA) nanoparticles, termed LND-PLGA/TPS/DSSR NP, was effective in triggering apoptosis in triple-negative breast cancer and was shown to increase the efficacy and decrease the toxicity of LND alone in a mouse model.^270^ In addition to these types of materials, other types of nanomaterials have been used for mitochondria-targeted therapies. A peptide-based “nano-boat” was found to trigger the mitochondrial apoptotic pathway and was effective in combination with radiotherapy *in vivo* to produce enhanced chemo-radiotherapy effects.^271^ Black phosphorus (BP) nanomaterials, assembled with TPP and PEG to form a BPQD-PEG-TPP mitochondria-targeting platform with good stability, dispersion, and negligible side effects, may be a promising strategy for mitochondria-targeted photothermal cancer therapy.^272^ A zero-valent-iron nanoparticle (ZVI-NP) can target both the tumor microenvironment and cancer cells, causing mitochondrial dysfunction, intracellular oxidative stress, and lipid peroxidation, leading to iron-toxic death of lung cancer cells.^273^

**Table 3:** Mitochondria-targeted drugs based on nanoparticles and nanomaterials.

Nanoparticles	Types	Loadable drugs or systems	References
Nanographene	Carbon-based nanomaterials	GFFP TPPa (Fe3O4, FA, TPP), ICG, *etc*.	258, 259, 260
MITO-Porter	Lipid nanomaterials	Adriamycin, pi-extended porphyrin-type photosensitizer, *etc*.	261, 262, 263
Triptolide-liposome	Lipid nanomaterials	SS-TP LPs (amphiphilic stachydrine–octadecane conjugate), *etc*.	264
PLGA	Polymeric nanomaterials	PLGA-PTX (PTX), *etc*.	266
TPP	Cationic nanomaterials	TPP-HCPT (HCPT), TPP-CNDs-S-CPT (CNDs, S–S, CPT), LND-PLGA/TPS/DSSR NP (LND, PLGA, TPP-TPGS, DSSR), *etc*.	257
			268
			269
			270
Black phosphorus	Phosphorus nanomaterials	BPQD-PEG-TPP (TPP, PEG)	272
Zero-valent-iron	Metallic plasmonic nanomaterials	ZVI-NP	273

A number of challenges remain in the research of nanomaterials targeting mitochondria, including issues of nanoparticle stability, selective targeting, biocompatibility, metabolism, and excretion of nanoparticles *in vivo*. Overall, nanomaterials targeting mitochondria are a promising therapeutic strategy, but are still in the research stage. With the continuous progress of science and technology, it is believed that such nanomaterials are expected to become one of the effective tools for future tumor therapy.

## Conclusions

Mitochondria play a crucial role in the occurrence and development of tumors, leading to genetic and metabolic changes in tumor cells. Mitochondrial dysfunction and metabolic disorders promote tumor growth, survival, and drug resistance, making mitochondria an attractive target for cancer treatment. The elucidation of these mechanisms and effects is ultimately aimed at finding better therapeutic targets to overcome various challenges in cancer treatment. Currently, there are relatively few drugs targeting mitochondria, and various potential therapeutic targets have been identified in different studies. Only a small portion has been used for the development of new drugs, and more targets are currently only in the theoretical phase. So, how to apply the potential targets in these studies to drug development is a problem that needs to be addressed. Furthermore, the targets for targeted mitochondrial therapy are not clear enough, and the specific targets and mechanisms of some drugs that have been used in clinical trials have not yet been elucidated. Clarifying the specific targets, mechanisms, and effects of these drugs can better guide their use, such as controlling dosage and usage and predicting drug side effects. Targeted mitochondrial drugs also have many side effects. Due to the different metabolic and energy requirements between tumor cells and normal cells, changes in mitochondrial structure and function may affect them differently, and thus, targeted mitochondrial therapy may have adverse effects on normal cells, leading to side effects. Due to the different metabolic and energy requirements between tumor cells and normal cells, targeted mitochondrial therapy may have adverse effects on normal cells, leading to side effects. There are currently some mitochondria-targeted drugs in clinical trials, but most are still in the early research stage. How to design effective, safe, and clinically promising mitochondria-targeted drugs is an important issue that needs to be addressed in the future. Furthermore, there is currently a lack of reliable mitochondrial detection methods. The commonly used mitochondrial detection methods still have some limitations, such as low detection accuracy and cumbersome operation. More accurate and convenient detection methods are required to support the research and clinical application of mitochondria-targeted therapy.

In summary, the complex interaction between mitochondria and cancer provides an exciting pathway for us to understand tumor biology and develop innovative treatment methods. Future research should focus on revealing the mechanism of mtDNA changes, analyzing mitochondria-mediated cell death regulation, and exploring metabolic reprogramming.

## CRediT authorship contribution statement

**Chen Huang:** Writing – original draft, Data curation, Investigation. **Zichuan Xie:** Writing – original draft, Investigation, Data curation. **Jiajin Li:** Writing – original draft, Software. **Chenliang Zhang:** Project administration, Supervision, Writing – review & editing, Funding acquisition, Conceptualization.

## Funding

This research was supported by the Sichuan Science and Technology Program (China) (No. 2024YFFK0343) and the 10.13039/501100001809National Natural Science Foundation of China (No. 32000533).

## Conflict of interests

The authors declared no competing interests.
